# Integrated Metabolomic and Microbial Analysis of Quality Dynamics in Channel Catfish (*Ictalurus punctatus*) Under Refrigerated and Frozen Storage

**DOI:** 10.3390/foods14071089

**Published:** 2025-03-21

**Authors:** Liwei Xia, Shun Zhou, Kaiqi Lian, Shengao Chen

**Affiliations:** 1 College of Life Science and Technology, Tarim Research Center of Rare Fishes, Tarim University, Alar 843300, China; 2 Yangtze River Fisheries Research Institute, Chinese Academy of Fishery Sciences, Wuhan 430223, China; 3 School of Biotechnology and Food Science, Anyang Institute of Technology, Anyang 455000, China

**Keywords:** channel catfish, storage, lipid oxidation, metabolomics, microbiomics

## Abstract

Channel catfish (*Ictalurus punctatus*) is a widely consumed freshwater fish known for its nutritional value but is highly prone to spoilage. This study investigated the quality changes of catfish muscle tissue under refrigeration and freezing through physicochemical, metabolomic, and microbial analyses. Results revealed that sensory scores decreased significantly during storage, with frozen samples maintaining similar scores to refrigerated ones after extended periods. Protein degradation and lipid oxidation, indicated by TVB-N and TBARS levels, were more pronounced during prolonged freezing. Metabolomic profiling identified 261 differential metabolites under long-term freezing, including elevated phosphatidylcholines, sphingomyelins, and disrupted amino acid pathways. Shifts in spoilage-associated microbial genera, such as *Pseudomonas*, and the correlations between microbial genera and specific metabolites, such as *Methylobacterium* with methylmalonic acid, highlighted microbial-driven spoilage processes. These findings provided a comprehensive understanding of quality deterioration during storage, guiding the development of enhanced preservation strategies for aquatic products.

## 1. Introduction

The channel catfish (*Ictalurus punctatus*), a freshwater fish native to North America, has gained popularity worldwide due to its unique meat quality and rich nutritional value [1,2]. With its rapid growth rate and adaptability, catfish has become a highly sought-after aquatic product, particularly in China [3]. Similar to other high-protein and high-moisture aquatic products, catfish are prone to microbial contamination and biochemical reactions during storage, which necessitates rigorous quality control measures to prevent the degradation of fish quality. Storage temperature is a vital factor affecting the preservation of aquatic products, with traditional methods such as refrigeration (4 °C) and freezing (−18 °C or lower) employed to extend shelf life [4]. Nevertheless, meat products, including catfish, still undergo certain changes during cold storage, despite the inhibitory effects of refrigeration and freezing on microbial growth and enzyme activity. These changes could be attributed to various factors, including the residual activity of enzymes, the growth of psychrotrophic microorganisms, and the physical and chemical transformations that occur in the product [5].

Extensive research has been devoted to investigating the storage conditions of channel catfish, with a particular emphasis on the impact of varying pre-chilling and frozen storage temperatures on their quality [6,7,8]. For example, the impact of chilling and freezing techniques, along with storage durations, on the histomorphology and physicochemical properties of channel catfish fillets has been investigated [6]. While these studies have primarily focused on changes in physicochemical parameters, such as pH levels, shear force, whiteness, and expressible moisture, the pivotal roles of microorganisms and the metabolic activities driven by inherent enzymes in defining the freshness level and shelf stability of aquatic products cannot be overstated [9,10]. Notably, the contributions of microorganisms and metabolite changes to catfish spoilage and deterioration remain poorly understood in real-world preservation scenarios and conventional cold-chain transportation. To address this knowledge gap, future research should aim to investigate both microbial and metabolite changes, providing a comprehensive understanding of the preservation dynamics of channel catfish under typical refrigeration and freezing settings.

In recent years, metabolomics has emerged as a powerful omics technology in food science research, enabling comprehensive analysis of changes in metabolic compounds within biological systems [11,12]. This approach enables both qualitative and quantitative evaluation of small molecule metabolites, revealing alterations in metabolic states, pathways, and networks under specific conditions. Furthermore, metabolomics is highly sensitive and can detect even minor changes in metabolic profiles that may be indicative of early quality degradation or subtle effects of storage conditions. This capability provides researchers with a powerful tool to understand the underlying metabolic mechanisms responsible for changes in food quality during storage. For instance, metabolomics could be employed to compare the effects of various preservation methods, such as refrigeration, freeze-drying, and vacuum packaging, on the metabolic profiles of aquatic products [13,14,15]. Additionally, the advent of 16S sequencing technology has enabled the monitoring of changes in microbial community structure and species abundance during processing and storage [16]. By analyzing the shifts in microbial community composition of aquatic products under different preservation conditions, 16S sequencing revealed the intrinsic relationships between microbial species and preservation effects, providing a scientific basis for optimizing preservation conditions [17,18]. Furthermore, integrating metabolomics and 16S sequencing approaches provides a more comprehensive understanding of the complex interactions between microbial communities, metabolites, and metabolic pathways, ultimately informing the development of effective preservation strategies for aquatic products.

This study investigated the physicochemical properties of channel catfish under two typical storage conditions: refrigeration at 4 °C and freezing at −18 °C. Key indices, including sensory scores, pH, thiobarbituric acid reactive substances (TBARSs), and total volatile basic nitrogen (TVB-N), were measured to evaluate quality changes during storage. Furthermore, systematic analyses of meat metabolomics and microbiome alterations under different storage conditions were conducted. The relationships between dominant microbial genera and the production of differential metabolites were elucidated through the construction of an association network. The findings of this study will provide robust evidence for the development of scientifically informed and rational preservation strategies, thereby promoting the sustainable development of the catfish industry and ensuring consumer health.

## 2. Materials and Methods

### 2.1. Sample Preparation

Ten fresh catfish (*I. punctatus*) with a mean weight of 1.31 ± 0.11 kg and a mean body length of 39.88 ± 2.17 cm were sourced from a local market in Wuhan, China. These fish were transported in a plastic container filled with aerated water and quickly delivered to the laboratory by car. Following their arrival at the laboratory, these fish were held in an aerated water tank for 2 h before being humanely euthanized by a swift blow to the head with a wooden stick. The catfish were then dissected, eviscerated, washed, manually de-skinned, and filleted into chunks (approximately 30 g each). The chunks were randomly assigned to four treatment groups: control (Con), refrigerated storage (R) at 4 °C for 48 h, and frozen storage at −18 °C for 2 weeks (FT) or 4 weeks (FF), with five muscle samples per group. Each sample was placed in a polyethylene bag and stored accordingly. Following treatment, a subset of the processed samples from each group was used for meat quality evaluation, while the remaining samples were stored at −80 °C for subsequent metabolomics and 16S rRNA sequencing analysis.

### 2.2. Sensory Evaluation

Sensory evaluation was performed using a modified method based on previous studies [10,19]. Following sampling, the fish meat stored at different temperatures was randomly coded to ensure blind evaluation. A trained panel of six assessors (three men and three women) with relevant experience in fish evaluation conducted the sensory assessment in a quiet, well-ventilated, and odor-free environment. The evaluation was based on the appearance, odor, color, and texture of the raw meat. The sensory scores were assigned as follows: 20–16, excellent quality; 16–12, good quality with no significant changes; and <12, significant decline in quality. The sensory rating scale is presented in Table 1.

### 2.3. Measurement of pH

Following a previously established method with minor modifications [20], 5 g of fresh catfish meat was homogenized with 45 mL of distilled water in a conical flask using a homogenizer (MHZ-01, JOANLAB, Huzhou, China). The homogenization conditions were set at 5000 rpm, 4 °C, and a duration of 2 min. After a 30-min incubation period at 4 °C, the mixture was filtered, and the pH of the filtrate was measured using an HQ40d pH meter (HQ40d, HACH, Loveland, CO, USA). Triplicate measurements were taken, and the mean pH value was calculated for analysis.

### 2.4. Measurement of TVB-N

The TVB-N contents in catfish meat samples were determined according to the method outlined in the National Standard of the People’s Republic of China (2016, GB 5009.228-2016) [21]. Specifically, 10 g of fresh catfish meat was homogenized with 75 mL of distilled water in a distillation tube and soaked for 30 min to ensure uniform dispersion. The mixture was then connected to a Kjeldahl nitrogen tester (Kjeltec8400, FOSS, Hillerød, Denmark) after adding 1 g of magnesium oxide, and TVB-N values were measured. The results were expressed as mg/100 g muscle with triplicate measurements performed for each group.

### 2.5. Measurement of TBARS

The TBARS values were determined according to a previously reported method with slight modifications [22]. Briefly, 5 g of catfish meat samples were homogenized with 20 mL of 10% trichloroacetic acid (TCA) solution, followed by centrifugation for 10 min (4000 r/min, 4 °C) with a centrifuge (TDZ4-WS, Changsha Xiangzhi, Changsha, China). The supernatant was filtered, and a 5 mL aliquot of the filtrate was mixed with 5 mL of 0.02 mol/L 2-thiobarbituric acid. The mixture was then heated in a boiling water bath for 20 min, rapidly cooled, and subsequently measured for absorbance at 532 nm (iMark680, BIO-RAD, Hercules, CA, USA), using 5 mL of TCA as a blank control. The results were quantified as mg of malonaldehyde (MDA)/kg muscle, with triplicate measurements performed for each group.

### 2.6. Microbiota Analysis

#### 2.6.1. DNA Extraction and Sequencing

Genomic DNA was extracted from the muscle tissue of catfish using a DNA kit (Omega Bio-Tek, Norcross, GA, USA) following the manufacturer’s protocol. The concentration and purity of gDNA were determined and the V3–V4 hypervariable region of the bacterial 16S rRNA gene was amplified using the primer pairs 341F and 806R. PCR amplification was performed using the following cycling parameters: initial denaturation at 94 °C for 2 min, followed by 30 cycles of 94 °C for 30 s, 55 °C for 30 s, and 72 °C for 45 s. A final extension was performed at 72 °C for 10 min, and the reaction was terminated at 4 °C. The PCR mixture (50 μL total volume) consisted of 25 μL 2 × ES Taq Master Mix (Dye), 2 μL 10 μM forward primer, 2 μL 10 μM reverse primer, 10 ng template DNA, and ddH_2_O to volume. To ensure reproducibility, PCR reactions were carried out in triplicate. The PCR products were subsequently isolated from a 1% agarose gel and purified with the Agencourt AMPure XP beads kit (Beckman Coulter, Allendale, NJ, USA). The concentration of the purified amplicons was measured and equimolar amounts of the purified amplicons were combined and sequenced using paired-end sequencing on the DNBSEQ-G99 platform (MGI, Shenzhen, China).

#### 2.6.2. Sequencing Data Analysis

The raw 16S rRNA gene sequencing reads were demultiplexed and quality-filtered using Trimmomatic (v0.36) and subsequently merged with FLASH (v1.2.0) following the parameters outlined in a previously established protocol [23]. Operational taxonomic units (OTUs) were clustered at 97% similarity using VSEARCH (v2.7.1). Alpha and beta diversity were calculated using QIIME2 (https://github.com/QIIME2/q2-feature-classifier, accessed on 23 May 2024) and visualized with R software (v4.1.0). Differences in relative abundances at the genus levels between groups were assessed using Wilcoxon tests, with significance set at *p* < 0.05.

### 2.7. Untargeted Metabolomics Analysis

#### 2.7.1. Metabolite Extraction

Catfish muscle samples (50 mg) were weighed precisely, and metabolites were extracted with 400 µL of methanol/water (4:1, *v*/*v*) containing 0.02 mg/mL of L-2-chlorophenylalanine. The mixture was cooled to −10 °C and homogenized at 50 Hz for 6 min using a tissue crusher (XU-JY96-IIN, Shanghai Xiniukeji, Shanghai, China). This was followed by sonication at 40 kHz for 30 min at 5 °C. Protein precipitation was achieved by incubating the samples at −20 °C for 30 min. Finally, after centrifugation at 13,000× *g* for 15 min at 4 °C (CT15RE, HITACHI, Tokyo, Japan), the supernatant was carefully collected for LC-MS/MS analysis. To monitor system stability and performance, a pooled quality control (QC) sample was prepared by mixing equal volumes of all samples. The QC samples were processed following the same procedures as the analytical samples and were injected periodically during the analysis.

#### 2.7.2. UHPLC-MS/MS Analysis

The processed samples were analyzed using an ultra-performance liquid chromatograph–electrospray ionization–tandem mass spectrometer (UPLC-ESI-MS/MS) system (UPLC, Shim-pack UFLC SHIMADZU CBM30A; MS, QTRAP^®^ 4500+, Shimadzu Corporation, Kyoto, Japan). An ACQUITY Premier HSS T3 Column (1.8 µm, 2.1 × 100 mm) was employed for separation. The mobile phase consisted of two components: solvent A (water with 0.1% formic acid) and solvent B (acetonitrile with 0.1% formic acid). The gradient elution program was as follows: 0–10 min, A/B (95:5 *v*/*v*); 10–12 min, A/B (5:95 *v*/*v*); 12–14 min, A/B (95:5 *v*/*v*); 14–15 min, A/B (5:95 *v*/*v*). An injection volume of 3 µL was used, with the column temperature kept at 40 °C. During the analysis, samples were maintained at 4 °C. The optimized operating conditions for the mass spectrometer were as follows: the ESI source temperature was set to 550 °C; the voltage was 5500 V for positive mode and −4500 V for negative mode. Ion source gas I (GS I) was maintained at 50 psi, gas II (GS II) at 60 psi, curtain gas (CUR) at 35 psi, and the collision-activated dissociation (CAD) parameter was set to medium. In the triple quadrupole (QTRAP) configuration, each ion pair was scanned and detected based on optimized declustering potential (DP) and collision energy (CE).

#### 2.7.3. Metabolite Analysis and Identification

Raw data were processed with XCMS (v3.12.0) in R for feature detection, retention time correction, and alignment. Batch effects were mitigated using quality control (QC) samples. Metabolites with a relative standard deviation (RSD) > 30% in QC samples were excluded from further analysis. Metabolite identification was performed using accurate mass and MS/MS data, matching observed *m*/*z* values and predicted molecular formulas (based on ppm error and adduct ions) against relevant databases, such as HMDB (http://www.hmdb.ca, accessed on 12 October 2024), massbank (http://www.massbank.jp/, accessed on 12 October 2024), KEGG (https://www.genome.jp/kegg/, accessed on 12 October 2024). Differentially regulated metabolites were determined using a variable importance in projection (VIP) score greater than 1 and an absolute log2 (fold change) exceeding 1. These metabolites were mapped to Kyoto Encyclopedia of Genes and Genomes (KEGG) pathways to facilitate biological interpretation using MetaboAnalyst 5.0 [24].

### 2.8. Statistical Analysis

The physicochemical indices were reported as mean ± standard deviation (SD). One-way ANOVA was used to determine significant differences (*p* < 0.05) among treatments. Spearman’s correlation analysis was conducted to identify associations between differential metabolites and core microorganisms.

## 3. Results and Discussions

### 3.1. Sensory Evaluation and pH Analysis

**Figure 1:**
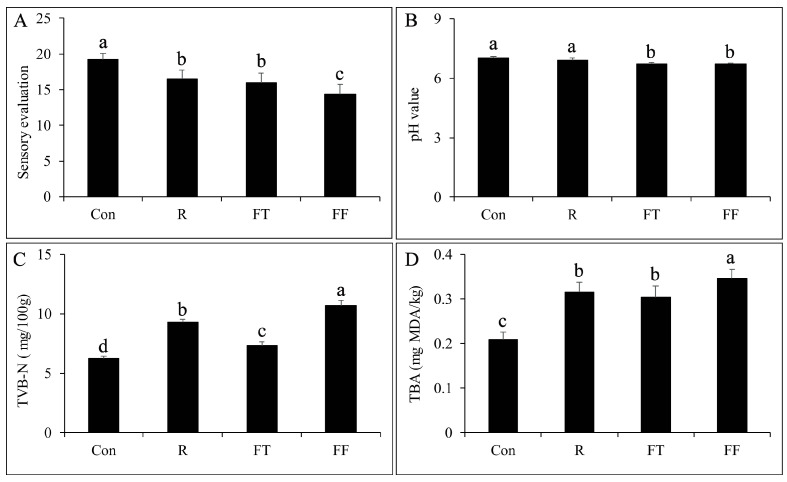
The alterations in sensory evaluation (**A**), pH (**B**), total volatile basic nitrogen (TVB-N) (**C**), and thiobarbituric acid reactive substances (TBARS) (**D**) of channel catfish (*Ictalurus punctatus*) muscle tissue under different storage conditions. Significant differences (*p* < 0.05) among groups are denoted by different lowercase letters.

The sensory evaluation scores for the muscle tissue of channel catfish under different storage conditions revealed notable variations. The control group (Con) exhibited the highest score of 19.2, indicating superior freshness. Compared to the control group, the R, FT, and FF groups showed significant decreases in sensory scores (*p* < 0.05), with values of 16.53, 16, and 14.4, respectively (Figure 1A). The decline in sensory scores with increased storage duration and lower temperatures suggests that both refrigeration and freezing impact the sensory attributes negatively, with prolonged freezing (FF) having the most pronounced effect. Despite the decline in sensory scores, all groups maintained scores above 12, indicating that these methods are still effective in preserving acceptable meat quality. The pH values exhibited a similar trend. The control group maintained a pH of 7.02, while the R group showed a slight, non-significant decrease to 6.9 (*p* > 0.05). Further significant reductions were observed in the frozen storage groups, with FT and FF recording pH values of 6.72 and 6.7, respectively (*p* < 0.05) (Figure 1B). The decrease in pH is likely due to the accumulation of lactic acid and other metabolites during storage, reflecting ongoing biochemical processes that can affect the quality of the fish [25].

### 3.2. TVB N and TBARS Analysis

The TVB-N levels served as a marker of protein degradation, with elevated values reflecting microbial activity and spoilage [26]. In the present study, the TVB-N values varied significantly among the treatment groups. The control group had the lowest TVB-N value at 6.2 mg/100 g, which is consistent with the expectation that fresh muscle tissue has minimal protein breakdown. In contrast, the R group at 4 °C for 48 h exhibited a notable increase in TVB-N to 9.1 mg/100 g (*p* < 0.05), suggesting the onset of protein degradation due to microbial and enzymatic activity. Both frozen storage conditions also showed a significant increase in TVB-N levels relative to the control group, with the FT group registering a TVB-N value of 7.4 mg/100 g and the FF group having a TVB-N value of 10.7 mg/100 g (*p* < 0.05). Although the values in the frozen storage groups were higher than those of the control group, they were still lower than the refrigerated group, which suggested that freezing may retard microbial and enzymatic activities to some extent. However, the increase in TVB-N with extended freezing time (FF) might indicate that prolonged frozen storage leads to a gradual breakdown of muscle proteins, possibly due to ice crystal formation and thawing during handling [27].

The TBARS values, indicating lipid oxidation, also increased with storage time [28]. The control group showed a low TBARS value of 0.21 mg MDA/kg, indicating minimal lipid oxidation. The R group showed a significant increase to 0.32 mg MDA/kg, indicating the initiation of lipid oxidation (*p* < 0.05). Both frozen storage treatments also demonstrated notable increases in TBARS values compared to the control group (*p* < 0.05). The FT group had a TBARS value of 0.3 mg MDA/kg, while the FF group exhibited the highest TBARS value of 0.35 mg MDA/kg. The increase in TBARS with longer frozen storage (FF) suggested that lipid oxidation continues slowly even at subzero temperatures. The higher TBARS value in the FF group might be attributed to the cumulative effects of oxidative processes during prolonged freezing, including oxidative damage caused by ice crystal formation or slight temperature fluctuations during storage [29].

Channel catfish is a popular freshwater aquaculture species for processing due to its high protein, moisture content, and nearly neutral meat quality. However, these characteristics make it susceptible to microbial contamination and spoilage during storage. Freezing and refrigeration are common preservation methods, but both can cause alterations in meat quality. This study found that sensory scores, pH values, TVB-N, and TBARS levels all changed under various storage conditions, but remained within acceptable limits [6]. These results suggested that both short-term refrigeration and long-term freezing are feasible for preserving catfish meat [30]. Freezing was more effective than refrigeration in reducing protein degradation (TVB-N), although extended freezing (FF) led to higher TVB-N values, indicating gradual protein breakdown. Similarly, while both freezing and refrigeration caused slight increases in lipid oxidation (TBARS), longer storage times led to greater oxidative damage. This observation aligns with a previous study demonstrating a steady increase in TBARS values in channel catfish (*Clarias lazera*) patties during controlled freezing-point storage [31]. Thus, while freezing generally preserves the quality of catfish muscle tissue, extended storage at subzero temperatures might accelerate both protein and lipid degradation, albeit more slowly than refrigeration.

### 3.3. Microbiome Analysis

#### 3.3.1. Microbial Diversity Analysis

**Figure 2:**
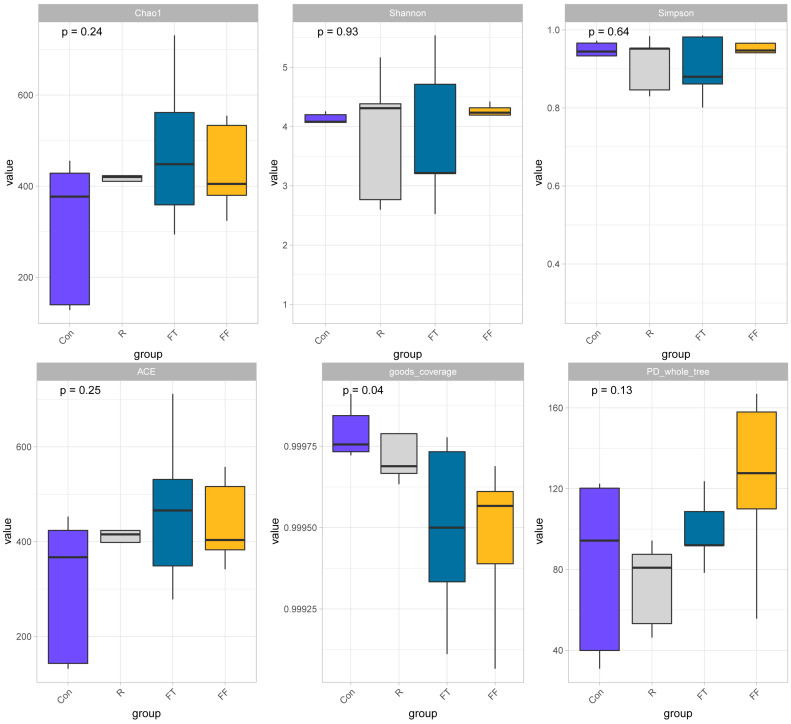
Microbial diversity analysis (alpha diversity metrics (Chao1, Shannon, Simpson, ACE, Good’s coverage, and PD whole tree)) of channel catfish (*Ictalurus punctatus*) muscle tissue under different storage conditions.

**Figure 3:**
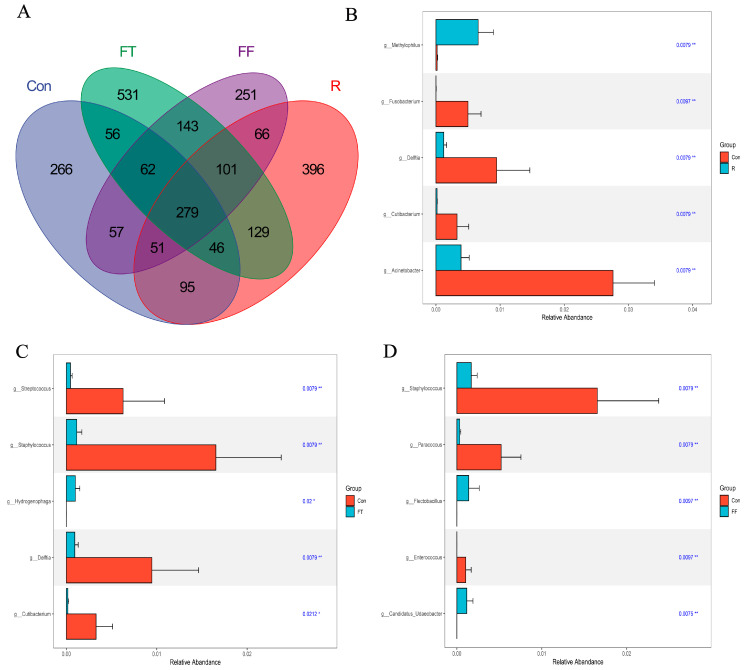
Microbial diversity analysis of channel catfish (*Ictalurus punctatus*) muscle tissue under different storage conditions. (**A**) Venn diagram of differential OTUs; (**B**–**D**) changes in the abundance of specific genera among the three comparisons (R vs. Con, FT vs. Con, and FF vs. Con). “*” indicates significant difference (0.01 < *p* < 0.05), “**” indicates extremely significant difference (*p* < 0.01).

To assess the impact of storage conditions on the microbial community structure of catfish muscle, 16S rRNA gene sequencing was employed. Alpha diversity indices, including Good’s coverage, Chao1, Shannon, Simpson, ACE, and PD whole tree, were calculated to evaluate microbial richness and diversity. The high Goods coverage index (99.9–99.99%) across all groups indicated sufficient sequencing depth and reliable detection of microbial communities. While Good’s coverage varied significantly among groups, no significant differences were observed in the other alpha diversity indices, suggesting that storage conditions influenced the extent of microbial diversity captured but did not substantially alter overall microbial richness or evenness (Figure 2). Venn diagram analysis revealed a core microbiome of 279 OTUs shared across all groups. The control group displayed the lowest OTU richness (912 OTUs), followed by the R group (1163 OTUs). Both frozen storage groups exhibited higher OTU richness, with the FT group (1347 OTUs) showing the highest (Figure 3A). These results highlighted that storage conditions significantly influenced the microbial community structure, with the highest diversity observed in the FT group, possibly due to fluctuations in environmental pressures during freezing. The slight decline in OTUs in the FF group compared to the FT group might be attributed to the selective survival of cold-resistant species or the inhibitory effects of prolonged freezing on certain microbes.

#### 3.3.2. Changes in Microbial Community Composition

**Figure 4:**
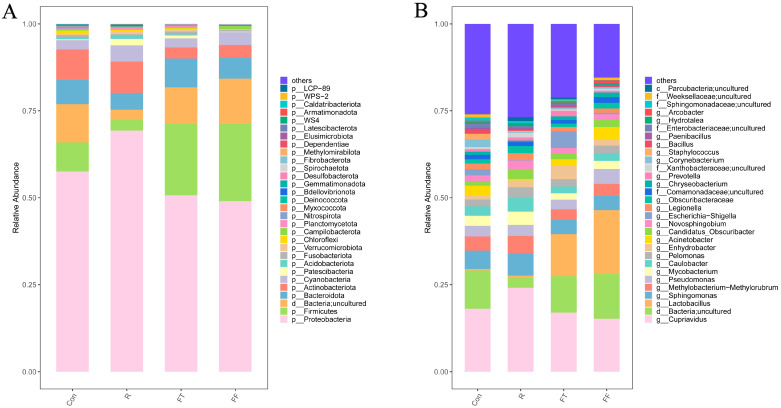
Microbial community composition at phylum (**A**) and genus (**B**) levels of channel catfish (*Ictalurus punctatus*) muscle tissue under different storage conditions.

At the phylum level, Proteobacteria, Bacteroidetes, Actinobacteriota, and Firmicutes dominated all groups, indicating that storage conditions did not drastically shift the dominant phyla (Figure 4A). At the genus level, the control group was characterized by the abundance of *Cupriavidus*, *Sphingomonas*, *Methylobacterium-Methylorubrum*, *Pseudomonas*, and *Mycobacterium* (Figure 4B). Refrigerated storage led to a significant increase in the relative abundance of *Acinetobacter*, *Cutibacterium*, and *Fusobacterium*. Conversely, *Methylophilus* was significantly reduced. The enrichment of *Acinetobacter* and *Fusobacterium* is consistent with previous studies that have identified these genera as key contributors to spoilage in refrigerated fish, likely due to their ability to thrive at low temperatures [32,33,34]. *Acinetobacter* is particularly noted for its adaptability to diverse environmental conditions, which allows it to effectively outcompete other spoilage microorganisms [35]. Additionally, *Fusobacterium* exhibited significant positive correlations with TVB-N and trichloroacetic acid in the muscle tissues of Mandarin fish (*Siniperca chuatsi*) [34]. In the frozen group, the abundance of *Staphylococcus*, *Delftia*, *Cutibacterium*, and *Paracoccus* was significantly elevated, suggesting that this storage condition might promote the growth of these genera [36].

Additionally, *Pseudomonas* was found to be ubiquitous across all storage conditions, consistent with previous reports highlighting its prevalence in aquatic environments and its association with spoilage in cold-stored fish [37]. This psychrotrophic bacterium thrives in refrigerated and frozen environments, where it can proliferate despite low temperatures. Notably, *Pseudomonas* showed increased abundance in the long-term freezing group (FF), aligning with findings from earlier studies demonstrating the survival and proliferation of *Pseudomonas* spp. in refrigerated and frozen conditions [38,39]. The increased abundance of *Pseudomonas* in the FF group likely contributed to the elevated TVB-N levels, which correlated with the noticeable quality decline in frozen catfish muscle tissue. These findings suggested that long-term freezing may not fully inhibit the growth of psychrotrophic bacteria like *Pseudomonas*. While freezing can slow down spoilage, it is not sufficient to completely prevent microbial growth. Prolonged frozen storage can lead to a gradual increase in microbial load, compromising the nutritional and sensory qualities of fish [40,41]. Therefore, it is essential to consider not only the duration of freezing but also the conditions under which freezing occurs to ensure optimal preservation. To further enhance the efficacy of frozen storage, pretreatment measures should be employed prior to long-term preservation. Techniques such as mild heat treatment, vacuum packaging, or modified atmosphere packaging can reduce microbial load, enzyme activity, oxidation, and microbial proliferation, thus enhancing quality and extending shelf life.

### 3.4. Metabolomic Analysis

#### 3.4.1. Differential Metabolites Analysis

**Figure 5:**
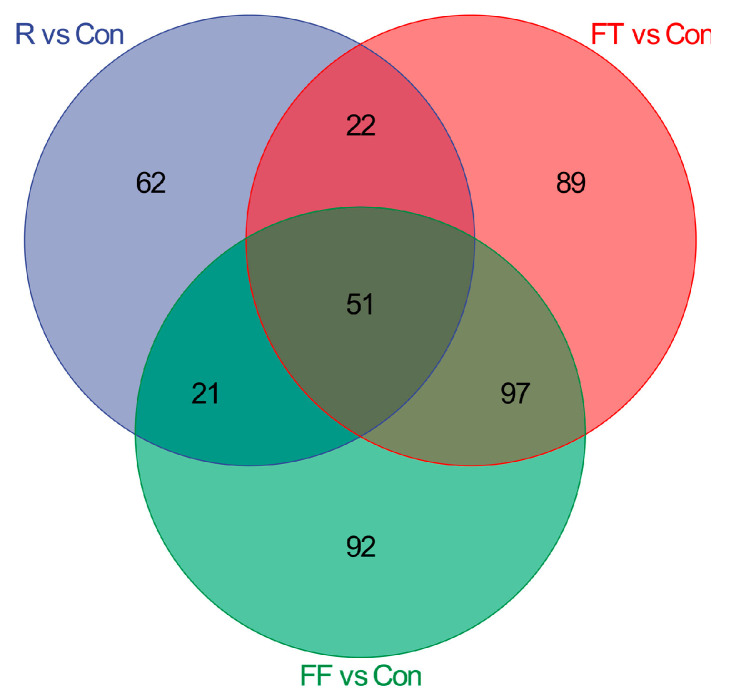
Venn diagram of differential metabolites of channel catfish (*Ictalurus punctatus*) muscle tissue under different storage conditions.

To gain deeper insights into the biochemical changes underlying the observed quality deterioration, a comprehensive metabolomic analysis was conducted. As shown in the Venn diagram in Figure 5, the number of differential metabolites (DMs) varied significantly across treatment groups compared to the control. In the R group, 156 DMs were identified, with 83 upregulated and 73 downregulated (Table S1). In the frozen storage groups, the FT group (2 weeks at −18 °C) exhibited 259 DMs, with 132 upregulated and 127 downregulated, while the FF group (4 weeks at −18 °C) had 261 DMs, with 146 upregulated and 115 downregulated (Tables S2 and S3). These results indicated that both refrigeration and freezing induced metabolic changes in catfish muscle tissue, with freezing over longer durations leading to a greater number of altered metabolites.

The metabolomic analysis also revealed significant alterations in lipid profiles across treatment groups, identifying differential metabolites such as phosphatidylcholines (PCs), phosphatidylethanolamines (PEs), sphingomyelins (SMs), and fatty acids (FAs). These lipid classes, particularly PCs and PEs, are crucial components of cellular membranes and are highly susceptible to oxidative stress. Peroxidation of these lipids resulted in the generation of lipid hydroperoxides and secondary oxidation products, which could further degrade membrane integrity [42,43]. The elevated abundance of SMs supported the hypothesis of oxidative stress, as these lipids play a role in membrane stability and could be targeted by reactive oxygen species (ROS) [44]. Additionally, the increased levels of FAs likely arose from the hydrolysis of phospholipids and triglycerides, a common consequence of oxidative and enzymatic processes in muscle tissue during storage [45].

The observed alterations in these differential abundant lipids (DALs) were indicative of oxidative deterioration. This process involved the generation of free radicals that initiated lipid peroxidation, leading to the breakdown of polyunsaturated fatty acids and the formation of secondary oxidation products, such as aldehydes and ketones [46]. These compounds contributed to off-flavors, reduced nutritional quality, and ultimately, spoilage. The differential abundance of lipids across storage conditions highlighted the susceptibility of fish muscle tissue to oxidative alterations during post-harvest handling. The alterations of PCs, PEs, SMs, and FAs could serve as biomarkers for lipid oxidation, providing insights into the progression of quality deterioration.

#### 3.4.2. Metabolic Pathway Analysis

**Figure 6:**
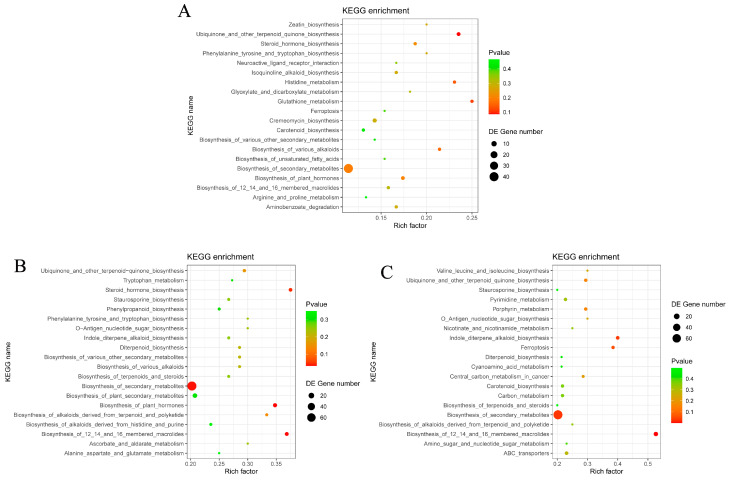
KEGG pathway analyses of differential metabolites among the three comparisons ((**A**): R vs. Con; (**B**): FT vs. Con; (**C**): FF vs. Con).

To elucidate the functional implications of the observed metabolite changes, KEGG pathway enrichment analysis was conducted for each treatment group compared to the control (Figure 6A–C). In the R group, the enriched metabolic pathways highlighted disruptions in amino acid metabolism (e.g., histidine metabolism and phenylalanine, tyrosine, and tryptophan biosynthesis) and secondary metabolite biosynthesis (e.g., alkaloid biosynthesis, plant hormone biosynthesis, and steroid hormone biosynthesis). These findings suggested that refrigeration triggered metabolic shifts, particularly affecting amino acid profiles and secondary metabolic regulation. For the frozen storage groups (FT and FF), the enriched metabolic pathways demonstrated more pronounced impacts on lipid metabolism and secondary metabolite biosynthesis. The regulation of lipid metabolism pathways, such as ubiquinone and other terpenoid-quinone biosynthesis, suggested increased lipid oxidation during frozen storage. Additionally, the activation of pathways involved in secondary metabolite biosynthesis, including alkaloid and terpenoid biosynthesis, might contribute to the development of off-flavors and odors [47,48].

The metabolomic analysis revealed distinct biochemical responses of catfish muscle tissue to refrigerated and frozen storage conditions. Refrigeration induced moderate changes in metabolite profiles, primarily reflecting early spoilage processes such as protein degradation and oxidative stress. In contrast, frozen storage, particularly in the FF group, caused more extensive metabolic alterations, likely due to prolonged freezing. The enrichment of pathways such as ferroptosis and porphyrin metabolism in the FF group suggested oxidative damage and heme degradation, potentially exacerbated by ice crystal formation and mechanical stress during freezing and thawing. Additionally, pathways related to ABC transporters and pyrimidine metabolism were enriched in the FF group, highlighting the metabolic effects of prolonged freezing on nucleotide metabolism and membrane transport processes. ABC transporters play a crucial role in the spoilage of aquatic products and are closely associated with the proliferation of spoilage-related bacteria, such as *Shewanella* [49]. The metabolites identified in these pathways serve as common spoilage markers and are indicative of quality deterioration in aquatic products.

### 3.5. Correlations Between Major Metabolites and Microorganisms

**Figure 7:**
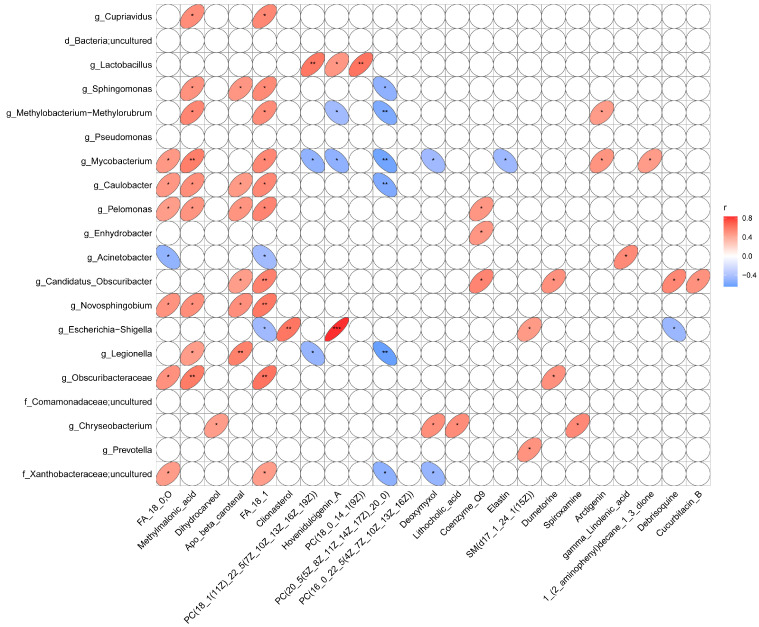
Spearman’s rank correlation analysis between major microbial communities and key metabolites in channel catfish (*Ictalurus punctatus*) muscle tissue during storage. Ellipse size indicates the magnitude of the correlation, and ellipse direction indicates positive or negative correlation. “*” indicates significant difference (0.01 < *p* < 0.05), “**” indicates extremely significant difference (0.001 < *p* < 0.01), “***” indicates very extremely significant difference (*p* < 0.001).

To investigate the relationship between major microbial communities and key metabolites in channel catfish muscle tissue during storage, a Spearman’s rank correlation analysis was conducted, and the results were visualized in a correlation heatmap (Figure 7). Methylmalonic acid exhibited strong positive correlations with *Obscuribacteraceae* and *Mycobacterium*. These findings suggested potential microbial contributions to the accumulation of methylmalonic acid, possibly linked to metabolic pathways involving amino acids or vitamin B12 metabolism, where methylmalonic acid may act as a byproduct [50]. Similarly, a significant positive correlation with *Methylobacterium-Methylorubrum* underscored the role of these genera in metabolite transformations during storage. Notably, *Novosphingobium* and *Obscuribacteraceae* showed robust positive correlations with FA 18:1, suggesting their involvement in lipid metabolism or transformation pathways. In contrast, FA 20:5 exhibited a significant negative correlation with *Legionella*, potentially reflecting microbial utilization of polyunsaturated fatty acids (PUFAs) under stress conditions such as oxidative or nutrient scarcity. The negative correlation between FA 20:5 and *Legionella* highlighted microbial lipid degradation processes during frozen storage [51]. This observation aligned with known mechanisms of microbial adaptation, where lipid catabolism supported resilience in low-temperature environments [42]. The reduction in PUFAs during storage suggested their susceptibility to oxidative and enzymatic degradation, mediated in part by microbial activity. The persistence of genera such as *Sphingomonas* and *Caulobacter* in frozen samples underscored their resilience and potential roles in lipid transformation under stress conditions. These psychrotolerant bacteria might contribute to the degradation or modification of lipids, impacting overall quality during prolonged storage. The correlations between specific microbial genera (e.g., *Pelomonas*, *Mycobacterium*) and metabolites like methylmalonic acid highlighted their potential as biomarkers for storage-induced quality changes. These relationships provided valuable insights into microbial dynamics and metabolic shifts that occur during fish storage, particularly in refrigerated and frozen conditions.

## 4. Conclusions

This study provided an integrative assessment of the quality dynamics of channel catfish under refrigeration and freezing storage, combining physicochemical, metabolomic, and microbial analyses. Refrigeration led to faster protein degradation, as shown by TVB-N levels reaching 9.1 mg/100 g within 48 h, while freezing limited these increases to 10.7 mg/100 g after 4 weeks. However, prolonged freezing induced significant lipid oxidation, with TBARS values increasing to 0.35 mg MDA/kg. Metabolomic analysis revealed substantial alterations in lipid oxidation products, such as phosphatidylcholines and sphingomyelins, alongside disrupted amino acid metabolism. These changes were linked to oxidative stress and enzymatic activity. Microbial profiling revealed spoilage-associated genera, including the enrichment of *Pseudomonas* in frozen samples and *Acinetobacter* in refrigerated samples, correlating with biochemical changes. Importantly, the study established correlations between key spoilage metabolites and microbial genera, such as *Methylobacterium* and methylmalonic acid, underscoring the role of microbial-driven biochemical transformations. These findings highlighted the need for refined storage practices that balance microbial suppression with biochemical stabilization. By integrating metabolomic and microbial analyses, this research provided actionable insights for optimizing catfish preservation, ensuring product safety, and extending shelf life in commercial settings.

## Figures and Tables

**Table 1:** The sensory analysis criterion.

Points	Color	Odor	Appearance	Texture
5	the color is bright and normal	it has a strong inherent aroma and no rancidity	the muscle texture is very clear and the tissue is firm	the muscle was elastic and the depression disappeared immediately after pressing
4	the color is normal	it has an inherent aroma and no rancidity	the muscle texture is clear and the tissue is firm	the muscles were elastic and the depression disappeared quickly after pressing
3	the color is dull	the inherent aroma is light and slightly fishy	the muscle lines are slightly clear and the tissue is not tight	the muscles were elastic and the depression disappeared very slowly after pressing
2	the color is very dull	the inherent aroma disappeared and there was a strong fishy smell	the muscle structure is partly loose	the muscles were less elastic and the depression disappeared very slowly after pressing
1	the color is dull and grey	it has a strong fishy smell	the muscle structure is loose	the muscle is inelastic and the depression barely changes after pressing

## Data Availability

The original contributions presented in this study are included in the article/Supplementary Materials. Further inquiries can be directed to the corresponding author.

## Supplementary Materials

The following supporting information can be downloaded at https://www.mdpi.com/article/10.3390/foods14071089/s1, Table S1: Differential metabolites between different treatments (R vs. Con); Table S2: Differential metabolites between different treatments (FT vs. Con); Table S3: Differential metabolites between different treatments (FF vs. Con).

## Abbreviations

The following abbreviations are used in this manuscript:

TVB-N	Total volatile basic nitrogen
TBARS	Thiobarbituric acid reactive substances
TCA	Trichloroacetic acid
MDA	Malonaldehyde
QC	Quality control
GS I	Ion source gas I
GS II	Ion source gas II
CUR	Curtain gas
CAD	Collision-activated dissociation
QTRAP	Triple quadrupole
DP	Declustering potential
CE	Collision energy
VIP	Variable importance in projection
RSD	Relative standard deviation
KEGG	Kyoto Encyclopedia of Genes and Genomes
SD	Standard deviation
